# Identification of essential genes associated with SARS-CoV-2 infection as potential drug target candidates with machine learning algorithms

**DOI:** 10.1038/s41598-023-42127-9

**Published:** 2023-09-13

**Authors:** Golnaz Taheri, Mahnaz Habibi

**Affiliations:** 1 Department of Computer and Systems Sciences, Stockholm University, Stockholm, Sweden; 2 Science for Life Laboratory, Stockholm, Sweden; 3 Department of Mathematics, Qazvin Branch, Islamic Azad University, Qazvin, Iran

**Keywords:** Machine learning, Gene ontology, Network topology

## Abstract

Severe acute respiratory syndrome coronavirus 2 (SARS-CoV-2) requires the fast discovery of effective treatments to fight this worldwide concern. Several genes associated with the SARS-CoV-2, which are essential for its functionality, pathogenesis, and survival, have been identified. These genes, which play crucial roles in SARS-CoV-2 infection, are considered potential therapeutic targets. Developing drugs against these essential genes to inhibit their regular functions could be a good approach for COVID-19 treatment. Artificial intelligence and machine learning methods provide powerful infrastructures for interpreting and understanding the available data and can assist in finding fast explanations and cures. We propose a method to highlight the essential genes that play crucial roles in SARS-CoV-2 pathogenesis. For this purpose, we define eleven informative topological and biological features for the biological and PPI networks constructed on gene sets that correspond to COVID-19. Then, we use three different unsupervised learning algorithms with different approaches to rank the important genes with respect to our defined informative features. Finally, we present a set of 18 important genes related to COVID-19. Materials and implementations are available at: https://github.com/MahnazHabibi/Gene_analysis.

## Introduction

As of January 2023, Severe Acute Respiratory Syndrome Corona Virus 2 (SARS-CoV-2), the virus that causes Coronavirus disease 2019 (COVID-19), has infected more than 650 million people worldwide and led to the deaths of more than 6.6 million people^1^. SARS-CoV-2 is a member of the Coronaviridae family of respiratory viruses and it is the third zoonotic coronavirus to emerge in the last 2 decades. SARS-CoV-2, in comparison to the other two coronaviruses, SARS-CoV (2002) and Middle East respiratory syndrome (MERS)-CoV (2012), has a lower rate of fatality and a higher rate of infection^2^.

Although there have been thousands of clinical trials, there are no approved medications for COVID-19 yet^3^. However, SARS-CoV-2 has a lower mutation rate than other coronaviruses. On the other hand, high genomic diversity is seen for SARS-CoV-2 both between individual patients and within the same virus class. This diversity enables the virus to adjust to a variety of hosts and circumstances within those hosts and is mostly related to disease development, drug resistance, and treatment results^4^. Therefore, even insignificant but continuous virus alterations and mutations would reduce the efficiency of vaccines or typically used drugs for COVID-19 treatments. Hence, collecting information about the virus’s evolution and pathology will be necessary to control the pandemic situation.

Many researchers are working to identify antiviral drugs and effective vaccines. Therefore, researchers are sharing their findings on SARS-CoV-2’s genome and evolution around the world. Some of these researchers are focusing on finding a therapy with the help of existing drugs using the drug repurposing method as a faster and less expensive approach^5^. Gene analysis is another useful method for drug repurposing and understanding different patients’ responses to the virus. Essential gene analysis can improve the understanding of SARS-CoV-2 data by recognizing the biological pathways of host cells affected by the virus. From the large amount of SARS-CoV-2 related data released, this kind of analysis can help to characterize possible drug targets and drug mechanisms of action^6^. As a result, to find an effective treatment, obtaining knowledge from data that characterized the SARS-CoV-2 host infection is a valuable approach.

Several genes associated with SARS-CoV-2, which are essential for its functionality, pathogenesis, and survival such as TNF, EGFR, and P53 have been recognized^7^. These genes crucial roles in SARS-CoV-2 infection and are considered possible therapeutic targets^8^. Ranking important and more relevant genes from all of the COVID-19 associated genes proposed by recent studies will help researchers focus on select sets of genes for further investigation. Developing drugs against these essential genes to inhibit their regular functions and associated physiological pathways could be a good approach to COVID-19 treatment.

In this work, we developed three unsupervised machine learning algorithms to specify important genes, which could help to identify effective COVID-19 treatments. For this purpose, we constructed two biological and Protein–Protein Interaction (PPI) networks corresponding to the COVID-19 related genes. Then, we defined eleven informative topological and biological features for each gene as a node in the network. We calculated three different scores with respect to our predefined features for each gene with respect to each algorithm. Afterward, we introduced the high-score genes in each algorithm with meaningful relationships to COVID-19 as candidate genes for more investigation. Finally, we presented a list of 18 genes that have been identified as top genes by at least two of our algorithms. These 18 genes could be targeted by some drugs like Abivertinib, chloroquine, and acetylcysteine which are approved as COVID-19 drugs.

## Related works

As an active area of machine learning research, feature selection tries to select a good subset of features to represent data. The eliminated features are mostly not informative; therefore, they are not considered for further analysis. Feature selection for supervised problems has been widely studied^9^. However, because class labels are unavailable to improve the search in unsupervised learning, feature selection for unsupervised problems is more complicated^10^. Feature selection for unsupervised problems such as clustering identifies a subset of features that builds informative clusters^10^. Therefore, feature selection for clustering reduces the data’s size and the run-time of learning algorithms and leads to more compact learning models with better generalization capability. The filter and wrapper are the two main approaches for unsupervised feature selection problems^11^. The filter approach assesses the significance of a specific feature subset primarily based on the inherent characteristics of the data, including variance, entropy, correlation, and local preservation, among other features. Filter approaches are often quick, scalable, and independent of any specific clustering algorithm. These filter methods are divided into univariate and multivariate techniques, which use some criteria to evaluate each feature and rank them by identifying and removing irrelevant features^11^. The univariate methods based on spectral analysis, such as Laplacian Score for Feature Selection (LSFS)^12^, follow the idea of modeling or identifying the local or global data structure using the eigensystem of Laplacian or normalized Laplacian matrices derived from an object similarity matrix. On the other hand, the multivariate methods jointly evaluate features, and the primary objective of these methods is to achieve feature selection or ranking rather than finding the cluster labels. In recent years, some multivariate methods under a new perspective called self-representation of features have been proposed. The assumption behind these methods is that a linear combination of appropriate features and a coefficient matrix with sparsity constraints can well approximate each feature. The Non-Convex Regularized Self-Representation (RSR)^12^ and Structure-Preserving Nonnegative Feature Self-Representation (SPNFSR)^12^ as an extended version of RSR, are two of the most used algorithms in this category of methods. The wrapper approach tries to evaluate the importance of a feature subset by considering its precision as the quality of the clustering result after applying a specific clustering method. Therefore, this approach depends on the selected clustering method and has a high computational cost^11^.

Determining associated genes with disease pathology is important in finding appropriate drugs. For COVID-19 related genes, infection-related genes, such as the inflammatory cytokines TNF$\alpha$, interleukins IL-1A, IL-1B, IL-R1, and IL-6, have been confirmed. Some verified genes are also related to certain diseases, such as heart disease, or some types of cancers, such as TP53 and EGFR, related to COVID-19^13^. There are extensive studies to identify essential genes related to COVID-19 disease, which can be used to identify therapeutic targets^14–16^. However, there is no comprehensive benchmark set of essential genes; therefore, comparing essential genes as the results of different methods is challenging. In this study, we introduced three sets of genes, each containing 50 high-score genes as essential, including a total of 131 genes. To investigate the 131 top essential genes, we compared these genes with four sets of essential genes known by independent algorithms with different approaches. The first set contains 93 genes related to disease pathology, which were identified by combining the biological and topological information of genes introduced by Habibi et al.^17^. This collection includes genes related to underlying diseases that play a vital role in the biological processes targeted by the virus. We denoted this set of genes as “Habibi”. The second set includes 130 related proteins HCoVs (SARS-CoV, MERS-CoV, HCoV-229E, and HCoV-NL63) which have been obtained with different experimental evidence. These host proteins are either direct targets of HCoV proteins or are involved in critical pathways of HCoV infection. We showed this set of genes with “VIPER”^18^. The third set includes 26 essential genes that can be introduced as drug targets. The authors of this study identified potential targets for repurposing based on Mendelian randomization. We denoted this set with “Erola”^19^. The fourth set contains 32 essential genes identified as the hub gene in the pathways related to COVID-19. We denoted this set with “Debmalya”^20^.

## Results and discussion

Identifying essential genes as drug targets plays a vital role in determining the mechanism of action of disease. Essential genes as drug targets are divided into three categories. The first category includes essential genes from the set of 29 identified virus proteins as SARS-COVID proteins^21^. The second category of essential genes includes numbers of host genes that directly interact with virus genes. Gorden et al.^21^ showed that 332 genes in the host cell interact with virus genes. The third category of essential genes includes host genes that do not directly interact with virus proteins but have been identified as host response genes, and disruption of these genes in the host cell can disrupt critical signaling pathways for the infection process^7^. This study only studied essential genes in the host cell as drug targets. We utilized three machine learning algorithms-LSFS, RSR, and SPNFSR, with different approaches to scoring 20,040 host proteins; then we selected 50 genes with the highest score as the top genes of each of these algorithms. This study aims to address the issue of identifying essential genes associated with COVID-19 as potential drug targets from two perspectives. Firstly, we utilized three distinct unsupervised machine-learning algorithms to solve the problem and analyzed the top 50 genes for each algorithm. We have presented a comprehensive list of these top 50 genes for each algorithm in Supplemental Table S1. Furthermore, we have listed the top 3 genes for each algorithm in Table 1 and provided evidence from other studies to support their potential as drug targets.

Secondly, we narrowed down our investigation to 18 genes that were identified by at least two of the three algorithms as promising drug targets. In Table 5, we have presented the potential drugs for these 18 genes, which have been confirmed by Drug Bank.

### Datasets

Identifying associated essential genes with disease pathology plays a major role in finding appropriate drugs. Thus, the starting point is to find suitable datasets to extract complete information about proteins and their relationships with COVID-19. For this purpose, we use the PPI network gathered in^17^. This dataset contains the physical interactions between proteins that are collected from the Biological General Repository for Interaction Datasets (BioGRID)^22^, Agile Protein Interactomes Data analyzer (APID)^23^, Homologous interactions (Hint)^24^, Human Integrated Protein–Protein Interaction reference (HIPPIE)^25^ and Huri^26^. All of the proteins in this dataset are mapped to universal protein resource (UniProt) ID^27^ and those proteins that could not be mapped to a Uniprot ID have been removed. This interactome contains 20,040 proteins and 304,730 interactions. We also use 1374 informative biological processes on the Gene Ontology (GO)^28^ that are reported by Habibi et al.^17^. These biological processes are linked to 332 human proteins, and Gorden et al.^21^ identified strong connections between these 332 human proteins and viruses. They define a biological process annotation as informative if it has two characteristics. (1) At least *k* proteins annotated with it. (2) Each of its descendants GO terms should have less than *k* proteins annotated with them. In this study, we set three for the value of *k*. We denoted these informative biological processes as IBPs. Among the 20,040 proteins, 9849 participate in the mentioned biological processes.

### Evaluation of high-score COVID-19 related genes

In this subsection, we studied the 50 top main genes with high-scores with respect to three different machine learning algorithms. Table 1 shows the three high-score genes resulting from three algorithms and the ranks of each gene in each algorithm. As mentioned earlier, these three algorithms have different approaches.

**Table 1:** Three high-score genes resulting from three algorithms and the ranks of each gene in each algorithm.

	LSFS ranks	RSR ranks	SPNFSR ranks
TNF	1	99	9
PTGS2	2	126	15
BCL2	3	102	77
NTRK1	76	1	55
APP	93	2	86
ELAVL1	138	3	111
CYP3A4	57	113	1
ABCB1	68	81	2
CYP2C9	81	79	3

The three genes, TNF, PTGS2, and BCL2, are identified as the three top genes with the highest scores selected by the LSFS algorithm. Studies have shown that TNF could be a key driver of inflammation in patients with severe COVID-19^29^. It could be targeted by existing immunomodulatory therapies. In^30^, the results of molecular docking analysis indicated that niacin showed effective binding capacity in COVID-19 and could help in COVID-19 treatment. One of the important pharmacological targets of niacin in COVID-19 was BCL2 and the other was PTGS2.

The three genes NTRK1, APP, and ELAVL1, are identified as the three top genes with the highest scores selected by the RSR algorithm. Studies on the NTRK1 gene showed that this gene is associated with the most important symptoms of severe COVID-19, and Fostamatinib, by targeting this gene, has been identified as a therapeutic drug for the control of acute respiratory distress syndrome (ARDS) in COVID-19 patients^31^. A recent study showed that the COVID-19 upstream regulators increased APP expression significantly. They revealed that molecular mechanisms of COVID-19 may lead to long-term neurological manifestations resulting from elevated APP expression^32^. Another study to prove the value of cellular RNA-binding proteins as therapeutic targets for COVID-19 treatment tested multiple drugs. Their results showed that one of these compounds targeting ELAVL1 caused a meaningful inhibition of SARS-CoV-2 protein production^33^.

The three genes CYP3A4, ABCB1, and CYP2C9, are identified as the three top genes with the highest scores selected by the SPNFSR algorithm. Authors in^34^ summarize medication updates for COVID-19 treatment in patients with an inflammatory state and their interactions with drug transporters. They showed CYP3A4, ABCB1, and CYP2C9 could be suitable targets for COVID-19 potential treatments.

**Figure 1:**
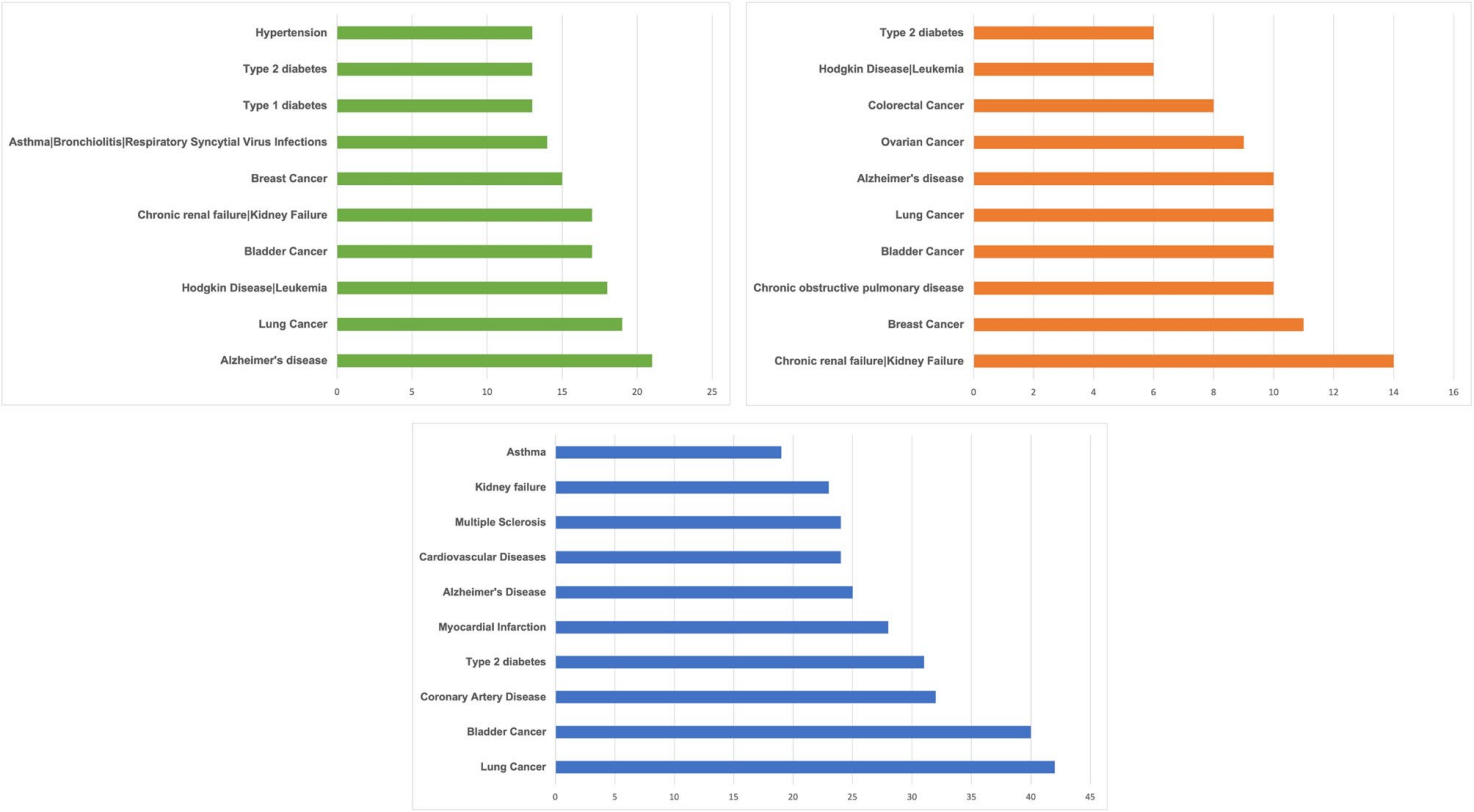
The list of top diseases and number of related disease genes for the LSFS (green), RSR (orange), and SPNFSR(blue) algorithms.

We also evaluated the list of significant diseases and associated pathways related to each of the 50 high-score genes for each of the algorithms. Figure 1 also shows that different types of cancer, autoimmune diseases, and diabetes have large numbers of common genes, with the top 50 genes resulting from the three algorithms. We also reported some of the significant disease pathway enrichments identified by the Database for Annotation, Visualization, and Integrated Discovery (DAVID) tools^35^. In the DAVID tools evaluation results, Fisher’s Exact p values are used to measure the gene enrichment in annotation terms. The geometric mean of members’ p values in a corresponding annotation cluster is also used to estimate the Group Enrichment Score. Table 2 shows the significant disease pathways with respect to the selected 50 high-score genes that are reported through the LSFS algorithm. These significant disease pathways like Hepatitis C, Influenza A, and Tuberculosis have significant p values. From a drug repurposing aspect, effective and most used drugs that target these common genes with selected 50 top genes (for both of the above-mentioned groups of diseases) could be possible COVID-19 treatments. Table 3 reports some of the significant disease pathway enrichments identified by the DAVID tool with respect to the selected 50 high-score genes that are reported through the RSR algorithm. These significant disease pathways like Hepatitis B and different types of cancers have significant p values. These pathways contain disease-associated genes that are reported through the RSR algorithm. Therefore, effective, and most used drugs for these diseases that target these common genes with selected 50 top genes could be possible COVID-19 treatments. Table 4 reports some of the significant disease pathway enrichments identified by the DAVID tool with respect to the selected 50 high-score genes that are reported through the SPNFSR algorithm. The significant disease pathways, such as Influenza A and Rheumatoid arthritis, exhibit significant p values and contain disease-associated genes that are identified through the SPNFSR algorithm. Hence, targeting the common genes in these pathways, including the top 50 selected genes, with effective and widely-used drugs for these diseases may lead to potential COVID-19 treatments and we recommend them for more comprehensive clinical studies.

**Table 2:** Top significant disease pathways resulting from the LSFS algorithm.

Term	Count	p value
Annotation cluster 1 (enrichment score: 12.121)		
hsa05168: Herpes simplex infection	20	4.20E$-$18
hsa05160: Hepatitis C	13	8.31E$-$11
Annotation cluster 2 (enrichment score: 7.794)		
hsa05145: Toxoplasmosis	19	8.34E$-$21
hsa05160: Hepatitis C	13	8.31E$-$11
hsa05164: Influenza A	13	1.92E$-$09
hsa05133: Pertussis	10	1.93E$-$09
hsa05161: Hepatitis B	12	3.66E$-$09
hsa05152: Tuberculosis	12	2.99E$-$08

**Table 3:** Top significant disease pathways resulting from the RSR algorithm.

Term	Count	p value
Annotation cluster 1 (enrichment score: 4.211)		
hsa04110: Cell cycle	9	6.28E$-$07
hsa05203: Viral carcinogenesis	8	2.09E$-$04
hsa05161: Hepatitis B	6	0.00176293
Annotation cluster 2 (enrichment score: 2.758)		
hsa05215: Prostate cancer	9	4.30E$-$08
hsa05205: Proteoglycans in cancer	9	2.24E$-$05
hsa05213: Endometrial cancer	5	2.63E$-$04

**Table 4:** Top significant disease pathways resulting from the SPNFSR algorithm.

Term	Count	p value
Annotation cluster 1 (enrichment score: 3.057)		
hsa05212: Pancreatic cancer	7	5.06E$-$06
hsa04010: MAPK signaling pathway	11	7.22E$-$06
hsa05160: Hepatitis C	8	3.28E$-$05
hsa05166: HTLV-I infection	10	5.25E$-$05
hsa05161: Hepatitis B	8	5.71E$-$05
Annotation cluster 2 (enrichment score: 4.663)		
hsa05164: Influenza A	11	2.36E$-$07
hsa05133: Pertussis	8	7.10E$-$07
hsa05152: Tuberculosis	9	2.57E$-$05
hsa05160: Hepatitis C	8	3.28E$-$05
hsa05161: Hepatitis B	8	5.71E$-$05

**Figure 2:**
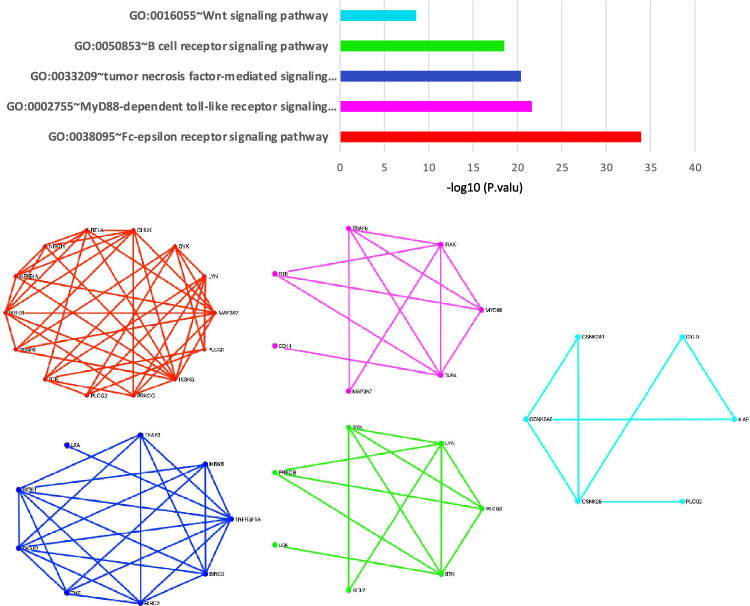
The biological processes with significant p values for top high-score genes through the LSFS algorithm.

**Figure 3:**
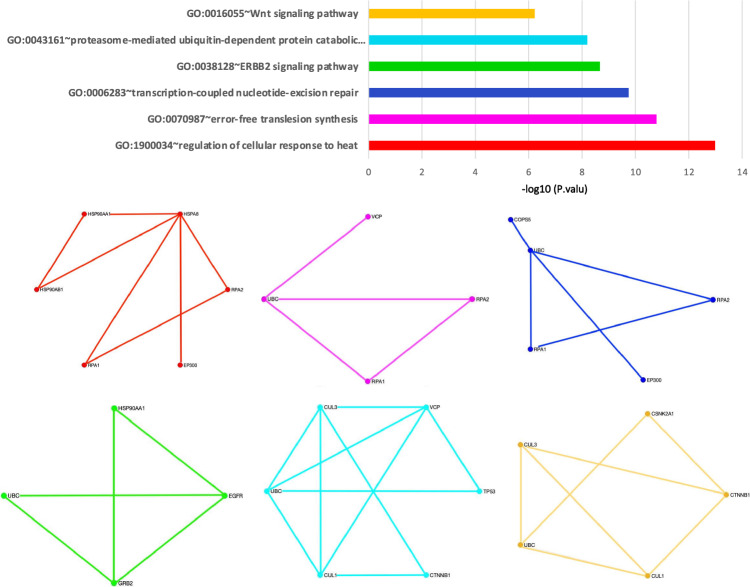
The biological processes with significant p values for top high-score genes through the RSR algorithm.

**Figure 4:**
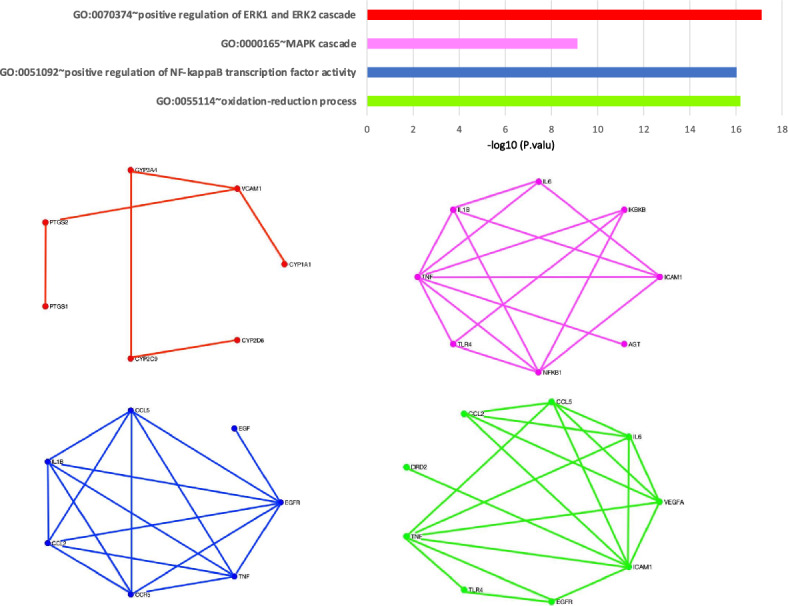
The biological processes with significant p values for top high-score genes through the SPNFSR algorithm.

In this study, we also studied the important biological processes in these high-score gene sets for each of the algorithms. We used the DAVID tool and identified five subsets of biological processes with significant p values as COVID-19 related modules. Figure 2 illustrates the p values of each of these modules and the connections between the genes of each of the modules for the LSFS algorithm. With the help of the DAVID tool analysis, it was identified that a part of the Fc-epsilon receptor signaling pathway (with a p value of 1.8 $$*$$ $$ E^{-15}$$) was a submodule in these high-score genes. Studies on this module showed that this signaling pathway is followed by the PI3k cascade, which is referred to as the COVID-19 associated pathway^20^. Studies also have shown that this module is associated with cytokine production in inflammatory diseases^36^. Another identified significant module is a part of the TLR signaling pathway as a MyD88-dependent pathway. In the MyD88-dependent pathway, the MyD88 protein recruits IRAK family proteins. The IRAK4 protein activates TRAF6 and this protein ultimately activates NF-$$\kappa $$B resulting in the production of excessive and dangerous inflammatory cytokines in patients with COVID-19^29^. Figure 3 contains six submodules with a significant p value from the DAVID tool for the RSR algorithm. We found that these modules have been identified in various studies related to COVID-19^37,38^. Figure 4 shows the value of each of these modules and the interaction network between them for the SPNFSR algorithm. We found that all of them have been cross-linked with important biological processes or COVID-19 related pathways. Treatment with Ang 1–7 is suggested in several studies. Ang 1–7 decreases the expression of intracellular signaling molecules such as the MAPK family (ERK1/2), which play an essential role in augmenting the inflammatory response^39^. Ang 1–7 also inhibits the NF-$$\kappa $$B signalings and reduces the expression of Ang II-induced ICAM-1 and VCAM-1. Treatment of COVID-19-affected patients with AT1R blockers (ARBs) may promote the ACE2/Ang 1–7 receptor with the reduction of proinflammatory cytokines and an increment in the level of anti-inflammatory cytokines^40^.

Finally, we studied the two sets of genes which includes the intersection (*C*) and the union (*U*) of the high-score genes for these three algorithms. We found that the vascular cell adhesion protein 1 (VCAM1) is reported across all three algorithms. VCAM1 is expressed on inflamed vascular endothelium in inflamed tissue and plays an important role in immune responses^27^. Also, it makes leukocytes migrate to locations of inflammation^27^. The systematic analyses showed that the increased expression of VCAM1 is related to COVID-19 disease severity and may contribute to coagulation dysfunction^41^. Set, *U*, denotes the union of these three algorithms’ results containing 131 genes. Among these 131 genes, five MOV10, RHOA, CSNK2A2, CSNK2B, and RIPK1 are identified as targets of the SARS-CoV-2 virus and have direct interactions with virus genes. Recent studies show that fostamatinib, as a potential drug for controlling COVID-19, can target two genes, CSNK2A2 and RIPK1^31^. Set, *U*, contains some infection-related genes, such as the inflammatory cytokines TNF$\alpha$, interleukins IL-1A, IL-1B, IL-R1, and IL-6, and other important genes, such as TP53 and EGFR. These essential genes associated with COVID-19 have been validated in clinical trials^13^.

**Figure 5:**
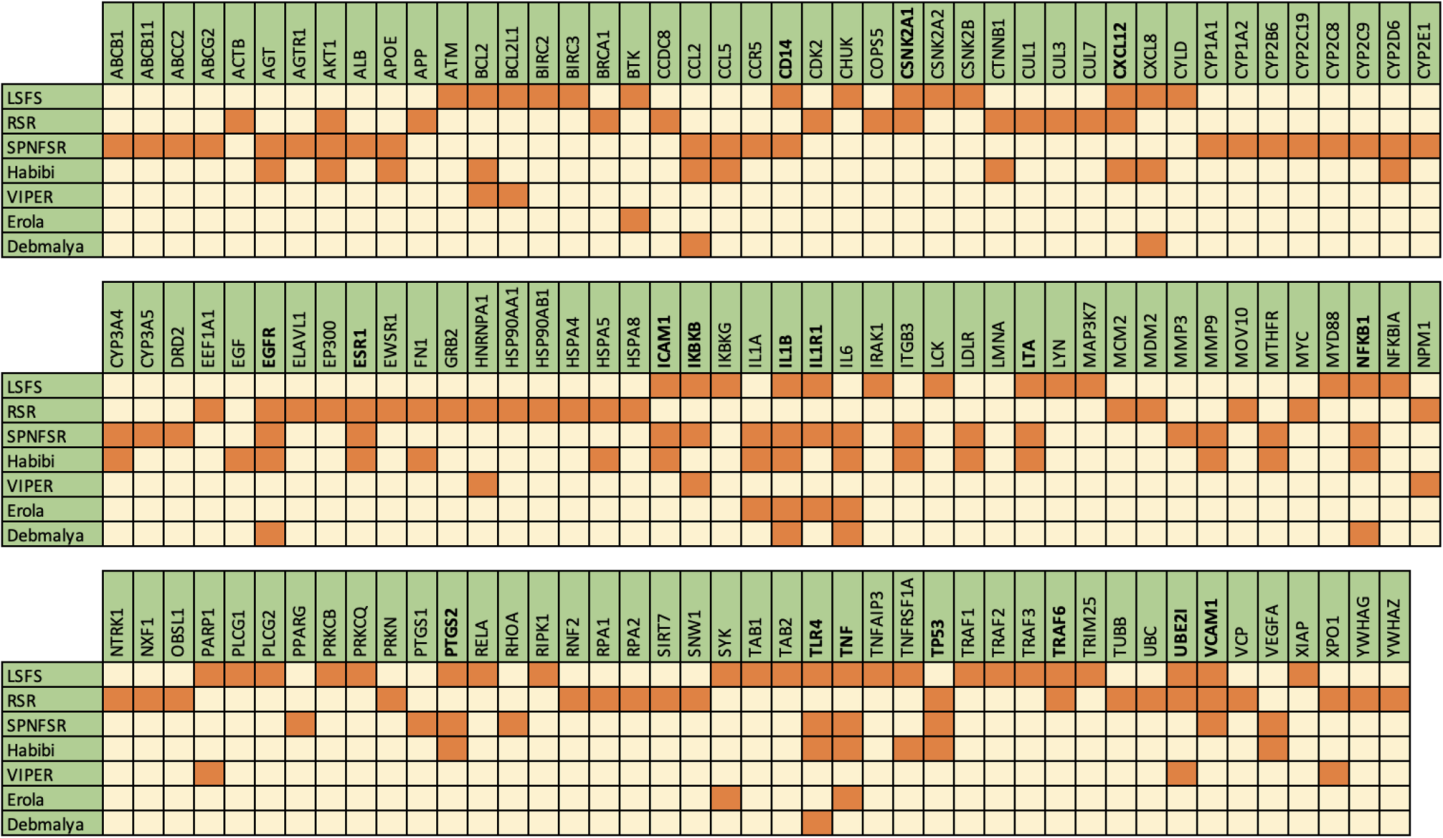
Comparison of high-score genes reported by LSFS, RSR, and SPNFSR, with the four mentioned algorithms.

**Figure 6:**
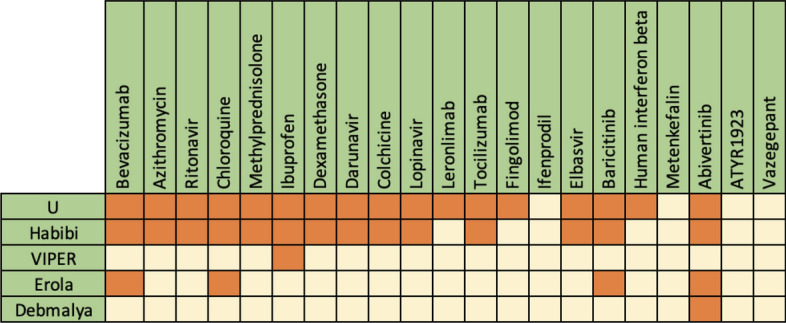
List of drugs approved by our high-score genes and related COVID-19 genes reported by other methods.

We compared the essential genes that are reported through four independent methods (Habibi, VIPER, Erola, Debmalya) with different approaches to essential genes resulting from our algorithms. Figure 5 compares the high-score genes obtained by LSFS, RSR, and SPNFSR, with the mentioned four algorithms. In this Figure, each gene detected through the mentioned algorithms is denoted with a darker color, and genes not reported through these algorithms showed with a lighter color. Figure 5 shows that 18 genes were identified by at least two of our proposed algorithms. Among these 18 genes, 14 genes as COVID-19 related genes are recognized by at least one of the four mentioned methods. We also find that high-score genes from the union of three algorithms, *U*, approve 17 drugs out of 21 experimental, unapproved drugs for COVID-19 reported in Drug Bank^31^. This set of drugs contains 69 experimental, unapproved drugs, and from these 69 drugs, 21 drugs have target information from host genes. Figure 6 shows the list of drugs approved by our high-score genes and related COVID-19 genes reported by other methods. Figure 6 shows that our high-score genes approved more experimental drugs for COVID-19.

### Evaluation of selected high-score COVID-19 related genes as drug targets

In the previous subsection, we evaluated the high-score genes obtained by each of our proposed machine-learning algorithms. The results of the previous subsection showed that each of these sets of genes with high scores has valuable genes as drug target potential. We also showed that 18 genes were confirmed by at least two of our algorithms, and more than 77% (14/18) of these genes were confirmed by at least one of the four studied methods. In the following, we analyze these genes in detail as genes with high potential in the COVID-19 treatment. Table 5 shows the complete list of these 18 genes and potential drugs for them. Each of these drugs is confirmed in Drug Bank as a potential drug in clinical trials or an approved drug for COVID-19 treatment^31^. In Table 5, the genes that have been confirmed in other studies or in^36^ to be associated with SARS-CoV-2 are shown in bold.

- TNF: TNF-$\alpha$ is one of the pro-inflammatory cytokines typically that is upregulated in acute lung injury and triggers cytokine release syndrome. The TNF-$\alpha$ facilitates SARS-CoV-2 interaction with angiotensin-converting enzyme 2 (ACE2). Therefore, the TNF inhibitors may perform as an effective therapeutic strategy for mitigating disease progression in severe SARS-CoV-2 infection^42^.
- LT-$\alpha$: As a member of the TNF family, it mediates a large type of inflammatory and antiviral responses. In COVID-19 patients, activated B cells produce IL-1, IL-6, IL-8, TNF, LT-$\alpha$, and other cytokines, which can aggravate the cytokine storm^43^.
- TLR4: The TLR family plays an important role in pathogen recognition and activation of innate immunity. TLR4 has a significant role in the pathogenesis of SARS-CoV-2, and its overactivation provokes a long or excessive innate immune response. TLR4 seems to be an appropriate therapeutic target in COVID-19 patients^31^.
- CXCL12: It plays a crucial role in diverse cellular functions like immune surveillance and inflammation response. The authors in^44^ showed that between mild and severe COVID-19 patients, significant differences were detected in plasma levels of CXCL12.
- ICAM1: It is an essential molecule in immune-mediated and inflammatory processes as a co-stimulatory signal for leukocyte trans-endothelial migration and T cell activation. The authors in^45^ showed that in COVID-19 patients, the levels of ICAM-1 were elevated and correlated with disease severity.
- IL1R1: It is an essential mediator involved in multiple cytokine-induced immune and inflammatory responses. The elevated levels of IL1R1 were reported in COVID-19 patients in recent studies^46^.
- PTGS2: It is responsible for the prostanoid biosynthesis involved in inflammation and mitogenesis. A recent study^47^ specifies the common key genes of COVID-19 and lung cancer through network analysis and one of these hub genes is PTGS2.
- NFKB1: It has a major role in the regulation of the early response to viral infection. Inappropriate activation of NFKB has been associated with several inflammatory diseases and upregulated levels of NFKB have been reported in COVID-19 patients^48^.
- IKBKB: It causes dissociation of the inhibitor and activation of NF-$\kappa$B, activated by numerous stimuli such as inflammatory cytokines and bacterial or viral products. Several studies confirmed the benefit of IKKs in weakening COVID-19. Therefore, IKBKB could be a potential therapeutic target for COVID-19 treatment^7^.
- IL1B: It is involved in inflammatory responses. It causes neutrophil activation, T-cell activation and cytokine production, B-cell activation, and antibody production. Patients with severe COVID-19 present high levels of IL-1B^7^.
- CD14: It collaborates with other genes to mediate the innate immune response to bacteria and viruses. It has been identified as a target candidate in the treatment of COVID-19^31^.
- TRAF6: As s member of the TNF family, it plays diverse roles in immune cells that regulate immune responses via control of inflammatory responses and recognition of innate immune signals. The SARS-CoV inhibits TLR-mediated signaling, reducing cytokine production during antiviral reactions by lowering the levels of TRAF3 and TRAF6 and then inactivating their downstream molecules, such as MAPK and transcription factors NF-$\kappa$B^49^.
- CSNK2A1: It can regulate numerous cellular processes, like apoptosis, transcription, and viral infection, and plays a major role in cancer progression and viral infection. It can be considered a potential drug target in cancers and COVID-19 therapy. Therefore, repurposing the cancer drugs to target CSNK2A1 could be a suggestion^50^.
- UBE2I: It is essential for nuclear architecture and chromosome segregation. The authors in^51^ hypothesized that interferences in the host nucleocytoplasmic trafficking of proteins partially depend on the SARS-CoV-2 relations with UBE2I.
- TP53: It works as a tumor suppressor, which means that it controls cell division by keeping cells from growing and dividing too fast or in an uncontrolled way. Researchers believe that SARS-CoV-2 will degrade the important tumor suppressor TP53, which will boost the virus’s ability to survive in host cells^7^.
- EGFR: It is a component of the cytokine storm which contributes to a severe form of COVID-19. Recent studies showed that SARS-CoV-2 depends on EGFR/ERK signaling and demonstrated EGFR inhibitors’ utility for COVID-19 treatment^52^.
- ESR1: It controls multiple cellular processes like growth, differentiation, and function of the reproductive system. The authors in^53^ revealed that estrogens interact with ESR1/2 receptors and can inhibit SARS-CoV-2-caused inflammation and immune response in host cells.
- VCAM1: It mediates the adhesion of lymphocytes, monocytes, eosinophils, and basophils to vascular endothelium. Recent studies indicated increased expression of vascular and inflammatory factors VCAM1 in COVID-19 lung tissue^54^.

**Table 5:** The list of shared genes that is identified by at least two of the proposed algorithms and the potential drugs for them which is confirmed in Drug Bank.

Gene name	Drug treatment
**TNF**	Infliximab
	Adamumab
**LT-**$\alpha$	Etanercept
**TLR4**	Cyclobenzaprine
	Golotimod
**CXCL12**	Tinzaparin
**ICAM1**	Nafamostat
**IL1R1**	Anakinra
**PTGS2**	Celecoxib
**NFKB1**	Dacomitinib
**IKBKB**	Acetylcysteine
**IL1B**	Anakinra
**CD14**	Atibuclimab
TRAF6	–
CSNK2A1	–
UBE2I	–
**TP53**	Zinc
**EGFR**	Abivertinib
**ESR1**	Zinc
**VCAM1**	Adalimumab

Genes with the approved drug have been shown in Bold.

## Conclusion

One of its main complications in COVID-19 patients is hyper-inflammation or the cytokine storm. Therefore, paying attention to inflammatory regulatory elements involved in SARS-CoV-2 infection can be the first step toward a comprehensive understanding of molecular regulatory mechanisms and the development of treatment strategies for COVID-19. The scientific community is trying to find new therapies for these inflammatory regulatory elements of COVID-19. For this purpose, researchers face a major challenge in identifying the fewest and most important COVID-19 related genes that could be used as potential drug targets. Numerous studies have been carried out to discover a suitable group of genes associated with COVID-19, and the results of these studies include a long list of genes, each of which could be important. It could be possible to identify effective drug targets by prioritizing these genes based on their topological and biological properties. We presented three machine learning algorithms (LSFS, RSR, SPNFSR) to prioritize COVID-19 related genes and organize these genes. The newly introduced algorithms are based on the feature selection method.

In the first part of this work, we defined 11 biological and topological features for each gene. The first four features, based on the centrality measure of each gene in the PPI network, are introduced as the topological features of the gene. We also built a COVID-19 related biological network. This network was a weighted network that fitted into a set of biological processes containing 332 proteins that were targeted by the virus. In this biological network, we have presented five features according to the topological characteristics of each gene as another measure for each gene. We also defined two other features for each gene in the PPI network. The first one was based on the number of drugs from the Clinical-Drug group that targeted the gene. The second one was based on the number of COVID-19 related signaling pathways that contain the gene. Then, with the help of three unsupervised machine learning algorithms, we assigned a score to these features. We assigned a score to each gene with the help of the topological and biological features of each gene and the value of each feature. We prioritized the set of genes based on these scores. In the result part of this work, we looked at the three high-scoring genes in each algorithm and discovered a direct link between these genes and COVID-19. We also evaluated the 50 top high-scoring genes of each algorithm with different measures. In the first measure, we evaluated the common genes between the list of 50 genes and disease genes for each algorithm. Our results show that these genes have the most in common with various types of cancer, diabetes, and autoimmune diseases. As another measure, we reported some of the significant disease pathways like Hepatitis C, Influenza A, and Tuberculosis with significant p values that contain disease-associated genes that have a lot in common with the list of 50 high-scoring genes. We also studied the biologically significant processes associated with these 50 high-scoring genes. We identified critical modules such as MyD88, Wnt, and MAPK, which have been linked to SARS-CoV-2 in multiple studies. Finally, we presented a list of 18 genes that have been identified as top genes by at least two of our algorithms. In Table 5 we showed the complete list of these 18 genes and potential drugs for them that were confirmed in Drug Bank as potential drugs in clinical trials or approved drugs for COVID-19 treatment. According to Table 5, our algorithms have identified many inflammatory related genes that play a key role in SARS-CoV-2 immunopathogenesis (such as TNF, IL1B, PTGS2, NFKB1, ICAM1, TP53, CD14, CXCL12, and EGFR) and this shows the high accuracy of our proposed method for gene analysis. We also compared our results with four different methods with completely different approaches and more than 77% (14/18) of the final set of genes were confirmed by at least one of the four studied methods.

## Materials and methods

In this section, we present a new method to identify essential genes associated with COVID-19 from two inputs: the PPI network and informative biological processes related to COVID-19. In the first step, we calculate four topological features for each protein in the PPI network. We also construct a biological network with respect to informative biological processes related to COVID-19 and calculate five informative features for each protein in the biological network. We also consider two biological features for each protein in the PPI network with respect to COVID-19 pathology. Then, for each protein, we generate a feature matrix $X=[x_{ij}]_{m\times n}$, where $x_{ij}$ represents the *j*-th feature for the *i*-th protein. In this step we used scaling to a range normalization technique to normalize our feature matrix.

In the second step, we use three unsupervised feature selection algorithms (LSFS, RSR, and SPNFSR) to calculate appropriate scores for each feature ($S_j$). Then, we define the Essentiality Score for each protein ($p_i$) as follows:

$$\begin{aligned} Essentiality \ Score \ (p_i) = \sum _{j=1}^n x_{ij} S_j \end{aligned}$$

**Figure 7:**
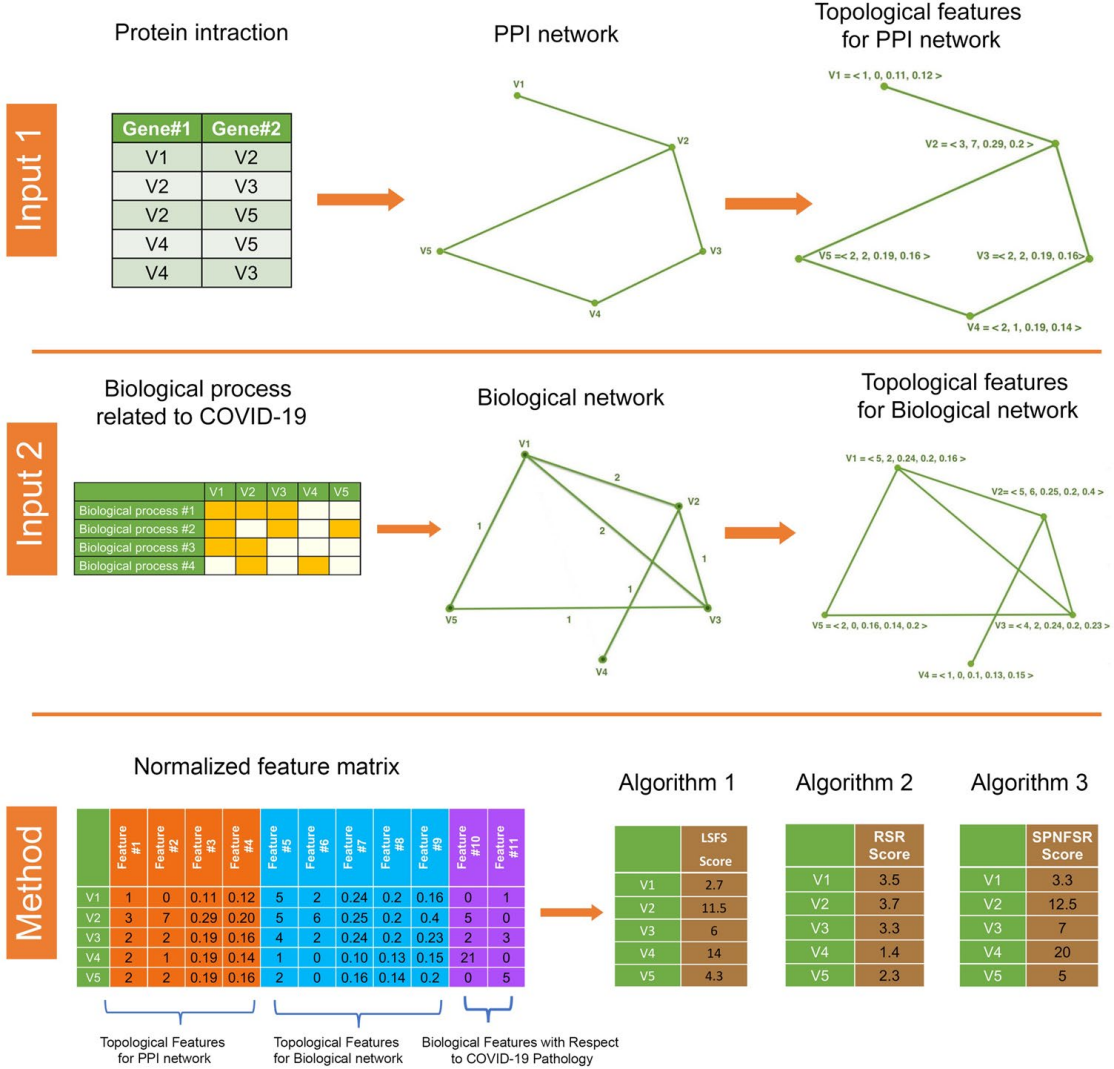
The workflow of the proposed methods.

The workflow of the proposed method to identify essential genes is illustrated in Fig. 7.

### Informative topological and biological features

In this section, we define informative topological and biological features for each protein in our dataset.

#### Informative topological features for PPI network

In a topological sense, a PPI network is modeled as an undirected graph $G=<V, E>$. Each protein in the PPI network is represented as a node, *v*, and the physical interaction between two proteins (*u* and *v*) is considered as an edge, *uv*. If *uv* is an edge of graph *G*, a node *u* is the neighbor of node *v*, and the set of neighbors of node *u* is represented by *N*(*u*). A path between *u* and *v* is determined as a sequence of distinct nodes $u=u_0, u_1,\ldots , u_n=v$ such that $u_{i}u_{i+1}$ is an edge of G. The length of a path is equal to the number of edges in this path. The distance between two nodes *u* and *v* is equal to the length of the shortest path between these two nodes, which is denoted by *d*(*u*, *v*). The following four informative topological features are defined for each node of the PPI network.

1. **Degree**: The number of neighbors of node *u* is defined as degree and denoted by *d*(*u*).
2. **Betweenness**: The betweenness centrality measure of each node *u* on graph *G* is defined as follows:
  1 $$\begin{aligned} B(u) = \sum _{v,w \in V} \frac{\psi _{v,w} (u)}{\psi _{v,w}}, \end{aligned}$$
  where $\psi _{v,w}$ denotes the total number of shortest paths between two nodes *v* and *w* and $\psi _{v,w}(u)$ shows the number of shortest paths between two nodes *v* and *w* pass through node *u*.
3. Pagerank: Another measure of centrality that is defined for each node *u* is the pagerank. This measure selects the score for each node *u* in the graph as a weighted contribution of all the scores assigned to the node *v* connected to *u* iteratively, as follows:
  2 $$\begin{aligned} PR(u) = (1-d) + d * \left[ \sum _{v, v \ne u}{ {\frac{1}{\sum _{w, w \ne v} 1} PR(v)} } \right] , \end{aligned}$$
  where *d* is a parameter between 0 and 1. *PR*(*u*) is the resulting score vector, whose *i*-th element is the score associated with node *u*. The larger score indicates the importance of the node according to its similarity with the other connected nodes.
4. Closeness: The closeness centrality measure for each node, *u*, is defined as follows:
  3 $$\begin{aligned} C(u) = \frac{|V|-1}{\sum _{v \in V} d(u,v)}, \end{aligned}$$
  where *d*(*u*, *v*) is the length of the shortest path between two nodes, *u* and *v*.

#### Informative topological features for biological network

In this section, we introduce a biological network with respect to 1374 informative biological processes related to COVID-19. This biological network is also modeled as a weighted undirected graph ${\mathscr {G}} = \langle \rho , \iota , \omega \rangle$. In this graph, each protein that participates in the mentioned biological processes is represented as a node. Two nodes, *u* and *v* are connected through an edge *uv* if two proteins participate in the same biological process. The weight of edge *uv* which is denoted by $\omega (uv)$, is the number of biological processes in which two proteins, *u* and *v*, participate. The length of the path in a weighted graph ${\mathscr {G}} = \langle \rho , \iota , \omega \rangle$, is the sum of the weights of edges encountered when passing through it. The length of a path is equal to the weight of edges in this path. The distance between two nodes *u* and *v* is equal to the weight of the shortest path between these two nodes, which is denoted by $d_\omega (u,v)$. The following five informative topological features are defined for each node of a weighted biological network.

1. Weight: The weight of *u* on weighted graph ${\mathscr {G}} = \langle \rho , \iota , \omega \rangle$, is defined as follows:
  4 $$\begin{aligned} {\mathscr {W}}(u) = \sum _{v \in N(u)} \omega (uv), \end{aligned}$$
2. Betweenness: The betweenness centrality measure of each vertex *u* on graph ${\mathscr {G}}$ is defined as follows:
  5 $$\begin{aligned} {\mathscr {B}}(u) = \sum _{v,w \in V} \frac{\psi _{v,w} (u)}{\psi _{v,w}}, \end{aligned}$$
  where the shortest path between two nodes, *v* and *w* is determined with respect to the length of the path in the weighted graph.
3. PageRank: Another measure of centrality that is defined for each node *u* is the PageRank. This measure selects the score for each node *u* in the graph as a weighted contribution of all the scores assigned to the node *v* connected to *u* iteratively, as follows:
  6 $$\begin{aligned} {\mathscr{P}}{\mathscr {R}}(u) = (1-d) + d * \left[ \sum _{v, v \ne u}{ {\frac{\omega (uv)}{\sum _{w, w \ne v} \omega (vw) }}{\mathscr {P}}{\mathscr {R}}(v)} \right] , \end{aligned}$$
  where *d* is a parameter between 0 and 1. ${\mathscr {P}}{\mathscr {R}}(u)$ is the resulting score vector, whose *i*-th element is the score associated with node *u*.
4. Closeness: The closeness centrality measure for each node, *u*, is defined as follows:
  7 $$\begin{aligned} {\mathscr {C}}(u) = \frac{|V|-1}{\sum _{v \in V} d_\omega (u,v)}. \end{aligned}$$
5. Entropy: Suppose that $W=[w_{ij}]$ be the weighted matrix correspond to weighted graph ${\mathscr {G}} = \langle \rho , \iota , \omega \rangle$ where
  8 $$\begin{aligned} w_{ij} = {\left\{ \begin{array}{ll} w(u_iv_j) &{} \quad \textit{if } \quad u_iv_j \in \iota \\ 0 &{} \quad \textit{ otherwise} \end{array}\right. } \end{aligned}$$
  For *j*-th node, we defined $P_j$ as follows:
  9 $$\begin{aligned} P_j = \frac{ \sum _{i=1}^N w_{ij}}{\sum _{j=1}^N{ \sum _{i=1}^N w_{ij}}}, \end{aligned}$$
  where $N=|\rho |$. We also define probability distribution vector $\pi = <P_1,P_2,\ldots ,P_N>$. Then the entropy of weighted graph is calculated as follows:
  10 $$\begin{aligned} Entropy ({\mathscr {G}})= -{\sum _{i=1}^N {P_i \log (P_i)}}. \end{aligned}$$
  The effect of each node, *u*, on network entropy is defined as follows:
  $$\begin{aligned} E(u)=|Entropy ({\mathscr {G}})-Entropy ({\mathscr {G}} -u)|, \end{aligned}$$
  where ${\mathscr {G}} -u$ is the weighted network that constructed with respect to removal of node *u* and its connected edges from network.

#### Informative biological features with respect to COVID-19 pathology

In this section, we also define two biological features for each protein in the PPI network with respect to COVID-19 pathology.

1. For the first feature, we use a set of experimental unapproved drugs in clinical trials for COVID-19 treatment that are available on the Drug Bank^31^. This set includes 708 drugs, of which 347 drugs have been studied clinically in more than one clinic. Among these 347 drugs, 213 drugs can target human proteins. This class of drugs is represented by Clinical-Drug. For each protein in the PPI network, the number of drugs approved through this protein is considered the first biological feature related to COVID-19 pathology.
2. For the second feature, we consider the most important signaling pathways related to COVID-19 (NF-$\kappa$B, Chemokine, Jak-STAT, P53, NOD-like, TNF, CAMP, RAS, Pap1, MAPK, PI3k-Akt, Toll-like(TLR)). The authors in^7^ proposed a comprehensive analysis for finding important pathways related to COVID-19 and they suggested these pathways as most important pathways related to COVID-19. For each protein in the PPI network, we calculate the number of these signaling pathways in which the protein participates.

### Unsupervised machine learning algorithms

Since the problem of finding the most important set of COVID-19 related genes is still an open question, it can be considered a problem without a response variable or exact answer. Therefore, to find an efficient answer, we used our defined informative features for our constructed COVID-19 related networks. Then, we employed three different unsupervised feature selection algorithms with different approaches to identify an efficient set of genes. It is worth mentioning that, in supervised learning methods, feature selection has been extensively studied. Due to the lack of information about class labels to help the search for relevant knowledge in unsupervised learning methods, selecting features is a significantly more difficult challenge^7^. Suppose $X=[x_{ij}]_{m\times n}$ represents the feature matrix that $x_{ij}$ represents the *j*-th feature of the *i*-th sample. We assign a feature vector $\overrightarrow{p_i }= <x_{i1},\ldots , x_{in}>$ to each sample and define the column matrix $F_j=[x_{1j}, \ldots , x_{mj} ]^T$ for the *j*-th feature. To find the appropriate score for each feature, we use three different unsupervised machine learning algorithms as follows. In the Supplemental file, we have described the detailed information and steps for each of these algorithms. We also added the detailed information about feature values for each algorithm in Supplemental Table S2.

#### Laplacian score for feature selection (LSFS)

Suppose that $S=[s_{ij}]_{m\times m}$ indicates the weighted matrix where $s_{ij}= e^{-\frac{|\overrightarrow{p_i } - \overrightarrow{p_j}|^2}{t}}$ if the euclidean distance between two feature vectors $\overrightarrow{p_i }$ and $\overrightarrow{p_j }$ is less than $\delta$. Also, suppose that $D=[d_i]$ is the diagonal matrix where $d_i= \sum _{k=1}^n s_{ik}$ and $L=D-S$ is the Laplacian matrix. The Laplacian Score for each feature, *j*, is calculated as follows:

11 $$\begin{aligned} S_j= \frac{\tilde{F_j}^T L \tilde{F_j}}{\tilde{F_j}^T D \tilde{F_j}}, \end{aligned}$$

where $J=[1,1,\ldots ,1]^T$ and $\tilde{F_j}=F_j - \frac{{F_j}^T D J}{J^T D J}J$. In this algorithm, we consider that $\delta$ = 5 and *t* = 100 respectively.

#### Non-convex regularized self-representation (RSR)

Suppose that $W^t$ indicates the weighted matrix and $w_j^t$ is the *j*-th row of $W^t$. Let $G_B^t=[g^t_{B,i}]_{m\times m}$ is the diagonal matrix where

$$\begin{aligned} g^t_{B,i}=\frac{1}{max\{ 2 \Vert \overrightarrow{p_i }-\overrightarrow{p_i } W^t \Vert _2, \varepsilon \}} \end{aligned}$$

and $G_W^t= [g^t_{w,j}]_{n\times n}$ is the diagonal matrix where $g^t_{W,j}=\frac{p}{2} \Vert w^t_j\Vert ^{p-2}_2$ $(0<p<1)$. For each $1\le t \le N$, the weighted matrix $W^{(t+1)}$ is calculated iteratively as follows:

12 $$\begin{aligned} W^{t+1}= ( (G^t_W)^{-1} X^T G^t_B X +\lambda I)^{-1} (G^t_W)^{-1} X^T G^t_B X, \end{aligned}$$

where *I* is the identity matrix and $\lambda >0$. Finally, to compute each feature’s weight using $S_j = \Vert w^j\Vert _2$ $(j=1, 2, \ldots , n)$ where $w^j$ denotes the *j*-th row of the weighted matrix *W*. In this algorithm, we consider that *p*=0.1, $\lambda$=1, *N*=60 and $\varepsilon$=0.01 respectively.

#### Structure preserving nonnegative feature self-representation (SPNFSR)

Suppose that $S_{m\times m}$ indicates the weighted matrix where $S=(|S|+|S^T |)/2$ shows the similarity of two feature vectors $\overrightarrow{p_i }$ and $\overrightarrow{p_j }$. Set two identity matrices $R_{m\times m}$, $Q_{n\times n}$. Compute matrix $L=(I-S- S^T+SS^T )$ and $M= X^T LX$. Suppose that $M= M^+- M^-$ where $M^+_{ij}=(|M_{ij}|+M_{ij})/2$ and $M^-_{ij}=(|M_{ij}|-M_{ij})/2$. The elements of weighted matrix *W* is calculated iteratively as follows:

13 $$\begin{aligned} W_{ij}= W_{ij} \frac{(\alpha M^- W + X^TRX)_{ij}}{((X^TRX+\beta Q+\alpha M^+)W)_{ij}}, \end{aligned}$$

where $\alpha \ge 0$ and $\beta \ge 0$ and two matrices *R* and *Q* as diagonal matrices updated iteratively as follows:

$$\begin{aligned} r_{ii}= & {} \frac{1}{max \{2 \Vert x_i - x_i W\Vert _2, \varepsilon \}},\\ q_{ii}= & {} \frac{1}{max \{ 2 \Vert w^i \Vert _2, \varepsilon \}}. \end{aligned}$$

where $\varepsilon$ is a very small constant. Finally, to compute each feature’s weight using $S_j = \Vert w^j\Vert _2$ $(j=1,2,\ldots , n)$ where $w^j$ denotes the *j*-th row of the weighted matrix *W*. In this algorithm, we consider that $\alpha$=0.05, $\beta$=0.05 and $\varepsilon$=0.01 respectively.

### Supplementary Information

Supplementary Information.

## Data Availability

The datasets generated and analysed during the current study are available in our GitHub repository, [https://github.com/MahnazHabibi/Gene_analysis].
