# Supplementary material for: Identification of essential genes associated with SARS-CoV-2 infection as potential drug target candidates with machine learning algorithms
Source: Sci Rep. 2023 Sep 13;13:15141. doi: 10.1038/s41598-023-42127-9 (PMC10499814; doi:10.1038/s41598-023-42127-9)
Supplement: Supplementary file 1 — Supplementary Information. [file 41598_2023_42127_MOESM1_ESM.pdf]

Identification of essential genes associated with SARS-CoV-2 infection as potential drug target candidates with machine learning algorithms-Supplementary File

Golnaz Taheri[1, 2, *,+] **and Mahnaz Habibi**[3, +]

[1]Department of Computer and Systems Sciences, Stockholm University, Stockholm, Sweden. [2]Science for Life Laboratory, Stockholm, Sweden.
[3]Department of Mathematics, Qazvin Branch, Islamic Azad University, Qazvin, Iran.
[*]Email:golnaz.taheri@dsv.su.se

[+]The authors wish it to be known that, in their opinion, the first and second authors should be regarded as Joint First Authors.

ABSTRACT

Severe acute respiratory syndrome coronavirus 2 (SARS-CoV-2) requires the fast discovery of effective treatments to fight this worldwide concern. Several genes associated with the SARS-CoV-2, which are essential for its functionality, pathogenesis, and survival, have been identified. These genes, which play crucial roles in SARS-CoV-2 infection, are considered potential therapeutic targets. Developing drugs against these essential genes to inhibit their regular functions could be a good approach for COVID-19 treatment. Artificial intelligence and machine learning methods provide powerful infrastructures for interpreting and understanding the available data and can assist in finding fast explanations and cures. We propose a method to highlight the essential genes that play crucial roles in SARS-CoV-2 pathogenesis. For this purpose, we define eleven informative topological and biological features for the biological and PPI networks constructed on gene sets that correspond to COVID-19. Then, we use three different unsupervised learning algorithms with different approaches to rank the important genes with respect to our defined informative features. Finally, we present a set of 18 important genes related to COVID-19. Materials and implementations are available at: https://github.com/MahnazHabibi/Gene_analysis.

keywords: Machine learning, Feature selection, SARS-CoV-2.

Supplementally Table S1

Selected 50 genes with the highest score for each of the three machine learning algorithms: LSFS, RSR, and SPNFSR, and their corresponding scores.

Rank	Genes	LSFS scores	Rank	Genes	RSR scores	Rank	Genes	SPNFSR scores
1	TNF	0.1953	1	NTRK1	7.43E+50	1	CYP3A4	0.1507
2	PTGS2	0.1525	2	APP	7.39E+50	2	ABCB1	0.1267
3	BCL2	0.0979	3	ELAVL1	7.23E+50	3	CYP2C9	0.0999
4	RELA	0.0811	4	CUL3	7.10E+50	4	CYP2C19	0.0801
5	IL1B	0.0755	5	GRB2	7.09E+50	5	CYP3A5	0.0744
6	TLR4	0.0701	6	TP53	7.07E+50	6	CYP2C8	0.0721
7	NFKB1	0.0687	7	EGFR	7.06E+50	7	CYP2D6	0.0702
8	IKBKB	0.0664	8	XPO1	7.04E+50	8	ALB	0.0698
9	RIPK1	0.0659	9	UBC	6.99E+50	9	TNF	0.0696
10	CHUK	0.0651	10	NXF1	6.98E+50	10	CYP1A2	0.0628
11	LYN	0.0634	11	HSP90AA1	6.97E+50	11	AKT1	0.0607
12	VCAM1	0.063	12	FN1	6.93E+50	12	IL1B	0.0573
13	TRAF6	0.061	13	COPS5	6.89E+50	13	IL6	0.0566
14	PARP1	0.0581	14	MCM2	6.87E+50	14	PTGS2	0.0559
15	CSNK2A1	0.0577	15	HSP90AB1	6.85E+50	15	NFKB1	0.0533
16	ICAM1	0.0473	16	ESR1	6.85E+50	16	TP53	0.0512
17	PRKCB	0.0449	17	VCP	6.84E+50	17	ABCB11	0.0495
18	TRAF2	0.0447	18	MOV10	6.83E+50	18	CD14	0.0492
19	IKBKG	0.0439	19	CTNNB1	6.83E+50	19	PPARG	0.0485
20	BTK	0.0432	20	NPM1	6.82E+50	20	ABCG2	0.0482
21	NFKBIA	0.042	21	CSNK2A1	6.81E+50	21	ITGB3	0.0481
22	ATM	0.0417	22	HNRNPA1	6.81E+50	22	APOE	0.0478
23	CD14	0.0408	23	HSPA8	6.80E+50	23	TLR4	0.0473
24	CXCL12	0.0395	24	MYC	6.79E+50	24	EGF	0.047
25	IL1R1	0.0376	25	ACTB	6.79E+50	25	IKBKB	0.0466
26	CSNK2B	0.0361	26	CUL7	6.79E+50	26	RHOA	0.0461
27	BCL2L1	0.0358	27	YWHAZ	6.77E+50	27	LTA	0.0454
28	UBE2I	0.0342	28	EWSR1	6.76E+50	28	CCR5	0.0454
29	TNFRSF1A	0.0305	29	VCAM1	6.76E+50	29	CYP2E1	0.0447
30	LTA	0.0289	30	RNF2	6.73E+50	30	CYP2B6	0.0444
31	BIRC2	0.0284	31	UBE2I	6.72E+50	31	CYP1A1	0.0437
32	MYD88	0.0281	32	CUL1	6.72E+50	32	MTHFR	0.0429
33	TNFAIP3	0.0271	33	CDK2	6.71E+50	33	IL1A	0.0428
34	LCK	0.0253	34	YWHAG	6.71E+50	34	ESR1	0.0425
35	CXCL8	0.0252	35	MDM2	6.71E+50	35	MMP3	0.0423
36	PRKCQ	0.0247	36	HSPA5	6.70E+50	36	CCL2	0.0416
37	TRAF1	0.0243	37	TUBB	6.70E+50	37	ICAM1	0.0414
38	SYK	0.0236	38	SNW1	6.70E+50	38	VEGFA	0.0414
39	BIRC3	0.0233	39	EP300	6.69E+50	39	CCL5	0.0409
40	PLCG1	0.0231	40	BRCA1	6.68E+50	40	AGT	0.0408
41	MAP3K7	0.023	41	LMNA	6.68E+50	41	DRD2	0.0407
42	IRAK1	0.0226	42	TRAF6	6.68E+50	42	VCAM1	0.0407
43	CSNK2A2	0.0219	43	SIRT7	6.68E+50	43	MMP9	0.0406
44	PLCG2	0.0214	44	PRKN	6.67E+50	44	IL1R1	0.0406
45	TAB1	0.0207	45	OBSL1	6.67E+50	45	CXCL12	0.0405
46	TAB2	0.0206	46	HSPA4	6.67E+50	46	PTGS1	0.0404
47	TRAF3	0.0202	47	RPA1	6.65E+50	47	LDLR	0.0403
48	CYLD	0.0194	48	RPA2	6.65E+50	48	EGFR	0.0402
49	TRIM25	0.0188	49	EEF1A1	6.64E+50	49	ABCC2	0.0401
50	XIAP	0.0187	50	CCDC8	6.63E+50	50	AGTR1	0.0399

Supplementally Table S2

The topological and biological feature values for three machine learning algorithms, LSFS, RSR, and SPNFSR.

It is worth noting that our proposed algorithms undergo a random initialization process. To obtain the final feature score, we conducted 50 runs for each algorithm and obtained the average feature values for each one. The average feature values were then used to rank the genes. The table provided showcases the results obtained from a single run of each algorithm.

	Informative topological features for PPI network				Informative biological features		Informative topological features for biological network				
Algorithms	Degree	Betweenness	Pagerank	Closeness	number of drugs approved	no. signaling pathways	Weight	Betweenness	PageRank	Closeness	Entropy
LSFS	0.8245	0.9854	0.773	0.0318	0.9922	0.9617	0.7774	0.9529	0.6724	0.5169	0.6802
RSR (E*+57)	0.0929	0.0933	0.2413	0.098	0.0875	0.097	0.0943	0.0898	0.096	0.099	0.091
SPNFSR	0.08	0.54	0.09	0.01	0.61	0.18	0.09	0.34	0.07	0.02	0.07

Supplementally Algorithm 1

The LSFS algorithm steps to calculate the score of each gene are described in Algorithm 1. In this algorithm, we considered $t = 100$ and $\delta = 5$ respectively.

Algorithm 1 Laplacian Score for Feature Selection (LSFS)

Require: Feature matrix $X = [x_{ij}]_{m \times n}$

Parameters t, δ

Let $\overrightarrow{p_i} = < x_{i1}, \dots, x_{in} >$ for each sample i

Let $F_j = [x_{1j}, \dots, x_{mj}]^T$ *for each feature j*

for $i \Leftarrow 1$ to m **do**

    for $j \Leftarrow 1$ to m **do**

        if $|\overrightarrow{p_i} - \overrightarrow{p_j}| < \delta$ **then**

            $s_{ij} = e^{-\frac{|\overrightarrow{p_i} - \overrightarrow{p_j}|}{t}}$

        else

            $s_{ij} = 0$

        end if

    end for

end for

$S = [s_{ij}]_{m \times m}$

$D = [d_i]_{m \times m}, where\ d_i = \sum_{k=1}^{m} s_{ik}$

$L = D - S$

$J = [1, 1, \dots, 1]^T$

for $j \Leftarrow 1$ to n **do**

    $\widetilde{F_j} = F_j - \frac{F_j^T D J}{J^T D J} J$

    $\zeta_j = \frac{\widetilde{F_j}^T L \widetilde{F_j}}{\widetilde{F_j}^T D \widetilde{F_j}}$

    $LS\,(g_i) = \sum_{i=1}^{m} x_{ij}\, \zeta_j$

end for

Supplementally Algorithm 2

The RSR algorithm steps to calculate the score of each gene are described in Algorithm 2. In this algorithm, we considered that $\lambda = 1, p = 0.1$ and $\varepsilon = 0.01$ respectively.

Algorithm 2 Non-Convex Regularized Self-Representation (RSR)

Require: Feature matrix $X = [x_{ij}]_{m\times n}$
Parameters λ, p and ε

Let $\vec{p_i} =< x_{i1}, \ldots, x_{in} >$ for each sample i
Let $F_j = [x_{1j}, \ldots, x_{mj}]^T$ for each feature j
Let I is the identity matrix
Repeat
Compute diagonal matrices $G_W^t = [g_{w,j}^t]_{n\times n}$ where $g_{w,j}^t = \frac{p}{2} ||w_j^t||_2^{p-2}$
Compute diagonal matrices $G_B^t = [g_{B,i}^t]_{m\times m}$ where $g_{B,i}^t = \frac{1}{\max\{2 \|\vec{p_i} - \vec{p_i}W^t\|_2, \varepsilon\}}$
$W^{t+1} = [w_{ij}]_{n\times n}$ using equation $W^{t+1} = ((G_W^t)^{-1} X^T G_B^t X + \lambda I)^{-1} (G_W^t)^{-1} X^T G_B^t X$
t= t + 1
Until convergence
for j ⇐1 to n **do**
$\zeta_j = ||w^j||_2$
end for
for i ⇐1 to m **do**
RSR (g_i) = $\sum_{j=1}^{n} x_{ij}\zeta_j$
end for

Supplementally Algorithm 3

The *SPNFSR* algorithm steps to calculate the score of each gene are described in Algorithm 3. In this algorithm, we considered that $\varepsilon = 0.01,\ \alpha = 0.05$, β=0.05, $t = 100$ and $\delta = 5$ respectively.

Algorithm 3 Structure Preserving Nonnegative Feature Self-Representation (SPNFSR)

Require: Feature matrix $X = [x_{ij}]_{m\times n}$

Parameters α, β, δ, t

Let $\vec{p_i} =< x_{i1},\ldots,x_{in} >$ for each sample i

Let $F_j = [x_{1j}, \ldots, x_{mj}]^T$ for each feature j

for i⇐1 to m **do**

    for j⇐1 to m **do**

        if $|\vec{p_i} - \vec{p_j}| < \delta$ **then**

        $s_{ij} = e^{-\frac{|\vec{p_i}-\vec{p_j}|^2}{t}}$

        else

        $s_{ij} = 0$

        end if

    end for

end for

Initialize sets R_1, Q_1, W_1 as identify matrices and $N = 1$

Compute $L = (I - S - S^T + SS^T)$

Compute $M^+ = (|M_{ij}| + M_{ij})/2$ and $M^- = (|M_{ij}| - M_{ij})/2$and $M = M^+ - M^-$

Repeat

Compute matrix $W_{N+1} = [w_{ij}]_{n\times n}$ using Equation

$$W_{ij} = W_{ij}\frac{(\alpha M^- W + X^T R X)_{ij}}{((X^T R X + \beta Q + \alpha M^+)W)_{ij}}.$$

Update diagonal matrices R_{N+1} and Q_{N+1} using Equation

$$r_{ii} = \frac{1}{\max\{2\,\|x_i - x_i W\|_2, \varepsilon\}},$$

$$q_{ii} = \frac{1}{\max\{2\,\|w^i\|_2, \varepsilon\}}.$$

$N = N + 1$

Until convergence

for j ⇐1 to n **do**

    $\zeta_j = \|w^j\|_2$

end for

for i ⇐1 to m **do**

    SPNFSR $(g_i) = \sum_{j=1}^{n} x_{ij}\zeta_j$

end for
